# Surgery as a Double-Edged Sword: A Clinically Feasible Approach to Overcome the Metastasis-Promoting Effects of Surgery by Blunting Stress and Prostaglandin Responses

**DOI:** 10.3390/cancers2041929

**Published:** 2010-11-24

**Authors:** Marganit Benish, Shamgar Ben-Eliyahu

**Affiliations:** Neuroimmunology Research Unit, Department of Psychology, Tel Aviv University, Tel Aviv 69978, Israel; E-Mail: benishd@post.tau.ac.il

**Keywords:** surgery, perioperative period, immunosuppression, prophylactic interventions, natural killer cells, stress, breast cancer

## Abstract

Surgery remains an essential therapeutic approach for most solid malignancies, including breast cancer. However, surgery also constitutes a risk factor for promotion of pre-existing micrometastases and the initiation of new metastases through several mechanisms, including the release of prostaglandins and stress hormones (e.g., catecholamines and glucocorticoids). However, the perioperative period also presents an opportunity for cell mediated immunity (CMI) and other mechanisms to eradicate or control minimal residual disease, provided that the deleterious effects of surgery are minimized. Here, we discuss the key role of endogenous stress hormones and prostaglandins in promoting the metastatic process through their direct impact on malignant cells, and through their deleterious impact on anti-cancer CMI. We further discuss the effects of anesthetic techniques, the extent of surgery, pain alleviation, and timing within the menstrual cycle with respect to their impact on tumor recurrence and physiological stress responses. Last, we suggest an attractive perioperative drug regimen, based on a combination of a cyclooxygenase (COX)-2 inhibitor and a β-adrenergic blocker, which we found effective in attenuating immune suppression and the metastasis-promoting effects of surgery in several tumor models. This regimen is clinically applicable, and could potentially promote disease free survival in patients operated for breast and other types of cancer.

## 1. A Calculated Risk: Surgery and Metastatic Development

Surgery for the removal of breast cancer is currently a necessary therapeutic strategy to achieve full recovery. Nevertheless, much effort is invested in adjuvant therapy, as the occurrence of metastases is the main cause of death in breast cancer patients. The extent of the surgery depends on whether the tumor has metastasized to the axillary lymph nodes, which is the most accurate prognostic factor in breast cancer [1,2]. The importance of addressing the metastatic process is exemplified in the routine excision of sentinel lymph nodes during surgery, and the removal of all regional lymph nodes if malignancy is found [3,4].

Given that evidence is continuously accumulating to support the hypothesis that surgery is a facilitator of the metastatic process, it is critical to consider the alarming clinical notion that surgery is a double-edged sword—it is necessary and life-saving; yet, has deleterious long-term impacts, potentially increasing the risk of metastases. We believe that surgery is indeed a major risk factor for the spread, establishment, and growth of malignancy. Several mediating mechanisms have been suggested to underlie this alarming hypothesis, including both direct effects of surgery and surgery-related release of humoral factors on the malignant tissue, as well as indirect effects of surgery such as immune suppression. First, the surgical incision of the tumor, its blood vessels, and proximate tissue, were shown to promote shedding of tumor cells from the notoriously non-cohesive malignant tissue into the blood and lymphatic systems [5,6]. Secondly, the removal of the primary tumor was suggested to eliminate the distant anti-angiogenic effect associated with its presence (carried by factors such as angiostatin and endostatin), hence promoting the survival and the outbreak of microscopic cancer foci that were previously detained by limited blood supply [7]. In addition, the tissue damage and the subsequent pro-inflammatory response induced by surgery were shown to elevate pro-angiogenic factors and other growth factors (e.g., EGF) [8,9,10], further promoting metastatic growth. The neuroendocrine and prostaglandin responses to surgery were shown to directly facilitate tumor cell metastatic capacity. Lastly, surgery was repeatedly shown to cause immune perturbations and suppression of cellular immunity, which were causally linked to promotion of the metastatic process in animal studies [11,12], and were associated with poor prognosis in cancer patients [13]. Noteworthy, there are numerous variations on surgical procedures, and several aspects of surgery were characterized as risk factors for metastatic progression, including blood transfusion (particularly long-stored erythrocytes) [14], hypothermia [15], and the use of specific anesthetic/analgesic compounds and techniques [16]. Most of these aspects of surgery were suggested to trigger some of the above mechanisms (e.g., causing neuroendocrine responses and immune suppression), leading to accelerated metastatic progression.

Although subjected to suppression, cell-mediated immunity (CMI), including cytotoxic T lymphocytes (CTL) and natural killer (NK) cells, is or could become a critical factor in controlling the progression of most malignancies during the perioperative period. Even though the immune system had failed in preventing the growth of the primary tumor, it is now becoming acknowledged that it can still be effective in limiting minimal residual disease during the perioperative period [17,18,19]. In this context, disseminated tumor cells are outnumbered by immune cells, and the immunosuppressive effect of the primary tumor may dissipate after its removal [20,21]. However, surgery-induced suppression of CMI is seen in patients’ blood samples [22] and in most immune compartments in animal studies [12,23]. NK cells are a front defense line against newly exposed cancer cells given that they respond immediately without the need for prior antigen presentation by major histocompatibility complex (MHC) class II [24,25]. Also, many tumors reduce their expression of MHC class I as an escape mechanism from CTLs [26], which makes these tumor cells more susceptible to recognition and destruction by NK cells [27]. Interestingly, NK cytotoxic activity is decreased even before the surgical procedure, and continues to decrease following it [22]. Most importantly, NK suppression is associated with increased rates of metastasis in cancer patients [13,28,29]. In animal models, surgery was causally linked to decreased NK activity, increased organ-specific tumor cell retention, and reduced postoperative survival rates [23,30].

In this review, we will focus on the stress- and surgery-related humoral mediators that lead to CMI suppression and direct effects on the malignant tissue, promoting tumor metastasis. Specifically, we will describe the contribution of catecholamines (CA), prostaglandins (PG), corticosteroids and other hormones. Finally, we will describe how a relatively safe, inexpensive, and simple manipulation of these mediators during the short but critical perioperative period holds promise in attenuating the deleterious effects of surgery.

## 2. Prostaglandins and Their Impact on Tumor Progression

Prostaglandins are paracrine and endocrine lipid agents that mediate pain and inflammation, and were suggested to play a key role in breast cancer progression and metastasis [31,32]. Prostaglandin E_2_ (PGE_2_) is secreted abundantly by damaged tissue endothelial cells, by various types of cancer cells, and by tumor-infiltrating macrophages. PGE_2_ is recognized by four different subtypes of G protein-coupled receptors (EP1-4) [33], and activation of these receptors causes an elevation in PKA and cAMP that mediates immunocyte suppression [34,35]. PGE_2_ significantly inhibited cytotoxicity of NK cells, through their EP2 and EP4 receptors, similar to their inhibition of γδT lymphocytes [35]. Additionally, PGE_2_ promotes the expression of the inhibitory NKG2A receptor on human CD8^+^T lymphocytes [36], reduces CD4^+^ T cell survival [37], and prevents the activation of naïve CD8^+^T cells [38]. In a model of lung cancer, PGE_2_ was shown to promote the activity of T regulatory cells, which play a role in suppressing antitumor immune responses [39].

Using a model for breast cancer metastasis, we have previously shown *in vitro* suppression of NK activity by PGE_2_, and an *in vivo* decrease in NK cytotoxicity and in resistance to MADB106 mammary carcinoma metastasis in rats [40]. Importantly, COX-2, but not COX-1 inhibition (e.g., using etodolac and celecoxib), attenuated up to 60% of the deleterious effect of surgery on NK cytotoxicity and on lungs tumor retention [12,23,40].

The administration of COX-2 inhibitors was also reported to prevent progression of malignancies by preventing the direct effect of PG on tumor cells [41,42,43]. Prostaglandin E_4_ antagonist inhibited metastasis of murine mammary tumor cells, and the silencing of the E_4_ gene in these tumor cells reduced the number of spontaneous metastases *in vivo*, and decreased inhibitory MHC class I antigen expression by tumor cells, causing them to become more susceptible to recognition and lysis by NK cells [44].

Many tumors secrete PG, presumably to evade recognition and destruction by immunocytes [37,38,45]. Overexpression of COX-2 induces genomic instability in breast cancer cells [46], promotes the progression of ductal carcinoma *in situ* to invasive breast carcinoma [47], and correlates with the number of metastases in the bones, lungs, and brain [48,49,50]. *In vitro* studies demonstrated that the administration of COX inhibitors promoted mammary tumor apoptosis via caspase 3 and 9, as well as via mitochondrial pathway [51]. Furthermore, mRNA levels for vascular endothelial growth factor (VEGF) and COX-2 and tumor microvessel formation were markedly decreased [47,51].

Overall, the release of PG by tumor cells and by tumor-infiltrating macrophages can be considered a tumor escape mechanism (in relation to immune destruction) and a tumor growth-promoting strategy, given the effects of PGE_2_ described above. We believe that tumor cells that did not acquire these strategies perished or were actively destroyed by the host. Thus, these escape mechanisms can be considered a consequence of cancer auto-evolutionary processes, as are other tumor escape mechanisms. Most importantly, the use of COX-2 inhibitors should be considered as potential adjuvant therapy in breast cancer patients, potentially reducing postoperative cancer recurrence, as we have shown in a mammary adenocarcinoma model of experimental metastasis in rats, and others showed in different tumor models [12,23,40,52].

## 3. Physiological Stress Responses to Surgery and Their Impact on Tumor Progression

There is an established link between psychological factors, including stress and depression, and the progression of several types of cancer [53,54]. The physiological mechanisms via which these psychological factors may impact tumor progression are a major focus of the following section. They include the activation of the sympathetic nervous system and the consequent secretion of catecholamines (e.g., epinephrine and norepinephrine); the activation of the hypothalamic-pituitary adrenal axis and the release of adrenal corticosteroids; and initiation of a variety of other stress responses leading to the secretion of opioids and several pituitary hormones. When considering surgery for tumor removal, it is clear that the stress responses associated with it stem both from psychological and physiological origins, involving the above-mentioned mechanisms, as well as additional mechanisms that are surgery-specific. The latter results from the use of anesthetic compounds and from tissue damage, which characterize most surgeries, and the consequent perturbations in prostaglandins levels, cytokine balance, and other physiological measures.

### 3.1. The Impact of CA

Epinephrine and norepinephrine were shown to decrease the cytotoxicity of NK cells and other aspects of CMI. This effect is mainly carried out through activation of β-adrenergic receptors (β-AR), which are expressed by all immune cells, profoundly by NK cells and CD4^+^ T cells [55]. The stimulation of β_2_-AR on immunocytes activates adenylate cyclase, which leads to intra-cellular accumulation of cAMP and activation of protein kinase A. This results in an inhibitory effect on NK cells and T cells [21]. *In vitro* incubation of human immunocytes with β-AR-agonists, or the administration of these agonists or of adrenaline to rats, resulted in decreased NK cytotoxicity against syngeneic cancer cells, which was prevented by β-adrenergic blockers such as nadolol [56,57]. Addressing endogenous stress responses, the administration of β-blockers (propranolol or nadolol) was shown to attenuate the deleterious effects of behavioral stress and surgery on NK-cytotoxicity and on *in vivo* resistance to lungs tumor colonization in a mammary adenocarcinoma model of metastasis [15,23] Besides the direct effect on CMI, CA also modify the T_H_1/T_H_2 cytokine balance toward T_H_2 dominance, which commonly supports humoral immunity and suppresses CMI [58]. For example, exposure of activated T_H_l cells to a β_2_-AR agonist or to a cAMP analog inhibits their production of the pro-CMI IL-2 cytokine [59,60,61].

In addition to suppressing CMI, CA and other surgery-related stress factors were shown to promote the growth of metastases by directly impacting tumor cells. These effects were blocked by the beta-antagonist propranolol in several cancer models including 66cl4 mammary tumor and HeyA8 and SKOV3ip1 ovarian tumors in mice [62,63]. In humans, cancers of the breast, pancreas, ovaries, and colon were shown to express β-adrenergic receptors (e.g., MCF-7/Her2 cells, MDA-MB231) [64]. Physiological levels of epinephrine were shown to protect prostate and breast cancer cells from apoptosis via the β_2_-AR and PKA dependent phosphorylation of the pro-apoptotic protein BAD, and via the G-protein βγ subunits [65,66]. Thaker *et al.* have shown in an *in vivo* study that stress-induced release of CA can activate β_2_-AR on human ovarian carcinoma cells, leading to signaling through PKA and an increase in the expression of tumor VEGF mRNA. Consequently, these processes led to enhanced tumor vascularization and a more aggressive growth and spread of malignant cells [67]. Similar observations were shown in pancreatic cancer and other carcinomas [68,69]. A recent study suggested yet another pathway via which stimulation of the β_2_-AR can promote tumor growth: activation of FAK, an intra-cellular factor that is associated with protection of ovarian cancer cells from anoikis (death after separation from extracellular matrix), increased survival time of detached cells, facilitated their migration, and promoted their reattachment and colonization in secondary sites [70].

Her2, a member of the EGFR family, is overexpressed in many human epithelial cancers, including breast cancers, specifically, in 30% of patients with breast cancer. This abnormality is associated with aggressive tumor growth and poor prognosis [71]. A recent paper by Shi *et al.* [64] suggested that in breast cancer cells the activation of the β_2_-AR up-regulates the expression of Her2 receptors via the activation of STAT3 in the MCF-7/Her2^+^ cell line, thus providing another mechanism through which CA can promote the spread of breast cancer. This intracellular path also stimulates the secretion of several cytokines involved in tumor metastasis, including IL-8 and VEGF. Interestingly, Her2 overexpression induces both the release of epinephrine and the up-regulation of β_2_-AR in MCF-7/Her2^+^ cells. This can induce a positive feedback loop between Her2 and β_2_-AR activation, further promoting breast cancer progression.

In contrast to the theme presented above, Carie *et al.* [72] have shown that stimulation of the β_2_-AR resulted in the blockade of the intracellular Raf-1/Mek-1/Erk1/2 pathway, leading to inhibition of growth and survival of human MDA-MB-231 breast cancer cells. These findings, however, have limited generalizability thus far, as they were reported regarding a single tumor line.

Surprisingly, the third CA, dopamine, also modulates CMI, but evidence suggest that dopamine has opposite effects than the other CA on tumor angiogenesis. Stimulation of T cells D_4_-dopamine receptors inhibits T cell receptor (TCR) activation [73], and the expression levels of KLF2, the transcription factor that regulates dormancy in T cells, were elevated in these cells. Interestingly, stimulation of other dopamine receptors that are expressed by T cells (e.g., D_1_/D_5_, D_2_, and D_3_) inhibited T cell proliferation but had no effect on KLF2 expression. Some dopamine receptors were reported to elevate cAMP (*i.e.*, D_1_ and D_5_), while others decrease it (*i.e.*, D_2_, D_3_, and D_4_), and further studies are required to determine the role of the different receptors in other immune cell populations. In tumor cells, dopamine down-regulates tumor angiogenesis [74,75], and enhances the efficacy of the commonly used anticancer drug 5-fluorouracil in mice bearing breast or colon cancer [76]. Overall, the perturbations in the systemic and in tumor-associated levels of dopamine in the perioperative period are yet to be studied, as is the impact of dopamine on *in vivo* tumor progression, both at the cellular and at the systemic levels.

### 3.2. The Impact of Glucocorticoids (GC)

Glucocorticoids (GC) in pharmacological doses have long been used to suppress immune functions, and were repeatedly implicated in mediating immune suppression by behavioral stressors and by surgery. We and others have shown that cancer patients and patients awaiting surgery have higher cortisol levels than matched controls subjects [77,78], apparently due to numerous disease-related factors, including psychological distress. Moreover, postoperative evening levels were significantly higher compared to the preoperative evening levels, indicating the additional impact of the surgical procedure. Laparotomy was shown to increase corticosterone levels in animals during the first 24 hours, and this pattern correlated with the decrease in NK cytotoxicity following surgery [79]. Recently it was shown that treating human NK cells with dexamethasone (a synthetic steroid) caused a dose dependent reduction in their cytotoxicity against the human leukemia K562 line. This treatment also reduced the production of intracellular IFNγ and TNFα by NK cells, reduced the expression of the adhesion molecule LFA-1, the natural cytotoxicity receptor NKp30, and granzyme B levels [80], all of which are believed to underlie reduced NK cytotoxicity. A possible mechanism for these effects is the induction or repression of gene transcription by GC. These may include the induction of the gene histone deacetylase, which in turn results in histone deacetylation, DNA tightening, and reduced expression of CMI promoting genes such as IFNγ [81,82]. Furthermore, other cytokines are downregulated by GC (including IL-1β, IL-6, IL-8, IL-12), and the secretion of many chemokines is strongly suppressed. In addition, anti-inflammatory cytokines such as IL-10 and TGFα are up-regulated by GC, and the T_H_2 cytokine response is promoted [83]. It was found that GC can reduce the expression of IL-12 receptors on T cells, and obstruct the responsiveness to IL-12 via intracellular phosphorylation in the T_H_1 response [84,85]. Overall, many of these effects are believed to contribute to CMI suppression in patients subjected to pharmacological treatments with GC or their agonists, and potentially in stressed patients.

Nevertheless, several studies have suggested that, in contrast to pharmacological levels, physiological stress levels of GC do not significantly suppress NK activity *in vivo* [86], or promote tumor progression. Specifically, we were able to abrogate stress- and surgery-induced suppression of NK activity using manipulations that do not affect GC levels [30,87]. Ongoing studies in our laboratory addressing the effects of stress and surgery indicate that, relative to CA and PG, GC are a minor player in the *in vivo* suppression of NK activity and resistance to experimental metastasis [88].

In the clinical setting, one should consider both physiological and pharmacological doses of GC. Synthetic GC are routinely administered together with chemotherapy to breast cancer patients, to alleviate treatment-related allergic reactions and nausea. Disturbingly, in addition to the expected immune-suppression it may cause*, in vitro* studies indicated that GC-receptor activation up-regulated pro-survival genes and decreased chemotherapy-induced apoptosis in breast cancer cells [89,90]. For example, GC-receptor activation was shown to inhibit FOXO3a mediated apoptosis, a pathway which is active in leukemia and breast cancer cell lines [91,92]. On the other hand, there are other studies showing that GC actually suppress angiogenesis in prostate cancer, renal cell carcinomas, and hematopoietic malignancies, and suppress prostate cancer growth *in vivo* in mice [93,94,95].

## 4. Anesthesia, Pain Relief and Opiates, and Their Impact on Tumor Progression

General anesthesia (e.g., using ketamine, thiopental, or halothane) was shown to suppress immune functions and promote tumor metastasis in animal models and in patients [96,97,98,99,100]. These effects of anesthetic compounds could be carried out through multiple pathways, including their impact on immune cells, endocrine responses, and malignant cells. For example, ketamine is also a weak β-AR agonist, and therefore it can directly affect CMI through leukocyte β-AR. Additionally, anesthesia-induced hypothermia can trigger the release of CA and cortisol [101,102], and breast cancer cells respond directly to opioid analgesics that accelerate their growth [15,103].

In contrast to general anesthesia with or without systemic analgesia, we reported that spinal block, in addition to general anesthesia, attenuated surgery-induced immunosuppression in rats, and reduced the metastasis-promoting effects of laparotomy [104,105]. We ascribed these beneficial effects to the blockade of sympathetic activity and reduced neuroendocrine stress responses to surgery, known to occur in patients operated under regional anesthesia [106]. Following and in line with our results, recent clinical retrospective studies suggested that regional anesthesia (e.g., paravertebral or epidural anesthesia and analgesia), used in breast [104,107,108] and prostate [109] cancer surgeries, is associated with an up to three-fold reduced risk of cancer recurrence during a follow-up period of several years. Prospective clinical studies are needed to confirm the advantage of paravertebral block in breast cancer patients, and epidural block in prostate cancer patients. The use of local anesthesia was also reported to be beneficial in skin cancer patients compared to general anesthesia [98]. By and large, local or regional anesthesia can be applied in most types of cancer surgery including ovarian and colon.

Drug-induced pain alleviation is a critical component in post-surgical treatment, but could be both beneficial and deleterious to the patient. Pain was shown to suppress a number of immune parameters, including the proliferation and activity of NK cells and other lymphocytes, by inducing endogenous release of opioids and other stress responses [110]. Morphine and other opiates are often used perioperatively given their potent analgesic effects; however, these drugs can also cause severe side effects, including nausea, vomiting, respiratory depression and most importantly immune suppression. For example, administration of fentanyl to rats suppressed NK activity and increased retention of tumor cells in the lungs [111]. This effect on the immune system may be mediated by elevated levels of glucocorticoids and/or CA [112,113]. Administration of Morphine to healthy volunteers caused a decrease in NK cytotoxicity [114]. Beilin *et al.* compared three regimens of postoperative opioid analgesics in a prospective clinical trial, and found that an epidural mixture of opiate and local anesthetics best prevented postoperative immune suppression [100].

However, not all opiate drugs have the same impact on immune responses. In operated rats, fentanyl and morphine significantly reduced NK activity, while buprenorphine did not [115], and the perioperative administration of the opioid tramadol prevented the promotion of metastasis by surgery in animals, and increased post-operative NK activity in cancer patients [116,117]. Therefore, these drugs may be preferred over morphine and similar immune-suppressing agents for the treatment of postoperative pain.

Direct effects of opiates on tumor cells are also reported. Gupta *et al.* showed that morphine has a tumor promoting effect by stimulating angiogenesis. In their *in vivo* model of MCF-7 breast cancer xenografts, morphine administration stimulated the phosphorylation of MAPK/ERK and the activation of the survival signals Akt and Cyclin D [103]. Moreover, morphine elevated levels of COX-2 and PGE_2_ in SCK-mammary carcinoma cells, and the administration of the COX-2 inhibitor celecoxib attenuated the effects of morphine on angiogenesis and metastasis. Interestingly, in this study the combination of morphine and celecoxib was the most efficacious in inducing analgesia and increasing survival rates. With respect to postsurgical analgesia and well-being, the combination of opioids and COX-2 inhibitors and/or NSAIDs improves clinical postoperative pain control [118]. Is spite of the aforementioned studies implying a deleterious role for morphine, other studies showed that morphine decreased the expression and secretion of matrix metallopeptidases (MMP)-2 and MMP-9 by MCF-7 breast cancer cells. These MMPs are enzymes that promote cancer invasion and metastasis capacity by degrading the extracellular matrix. However, these effects of morphine were not carried through µ-opioid receptor, as naltrexone did not block them [119]. Others have shown that morphine inhibits hypoxia-induced VEGF secretion and tumor growth in mice [120]. Comparing these allegedly inconsistent studies is intricate, as different tumor models were used and the doses and administration routes of the drugs were different. The question regarding the effects of morphine administration on tumor progression is yet to be resolved. Nevertheless, the use of the morphine-sparing COX-2 inhibitors in combination with morphine is clinically advantageous when immune suppression and other side effects of morphine are considered [121,122,123]. Indeed, a recently published retrospective research showed that the intraoperative use of Ketorolac, a non-selective COX inhibitor, reduced the risk of breast cancer relapse, compared to other commonly used analgesics, such as sufentanil (opiate), ketamine (NMDA receptor antagonist), and clonidine (alpha-2 adrenergic agonist) [124].

## 5. Does the Extent of Surgery Impact Neuroendocrine, Immune, and Clinical Outcomes of Cancer Progression?

In general, it was suggested that the magnitude of the surgical trauma determines the extent of postoperative immune suppression [125]. In non-breast surgeries, many studies show that laparoscopic surgery is advantageous over open surgery, when considering postoperative pain, return to normal activity, cosmetic appearance, and the effect on the immune system [125]. These effects were associated with a weaker stress response and CA secretion after laparoscopy, compared to laparotomy [126,127,128]. A prospective study by Wichmann *et al.* showed that NK cells are less affected by laparoscopic surgery, but CTL and CD4^+^ T cells are equally depressed after conventional open or laparoscopic surgery for colorectal diseases [129]. In mice we showed that paw amputation, which caused less bleeding and tissue damage than laparotomy and was conducted during a significantly shorter exposure to anesthesia, also resulted in a significantly lesser suppression of NK activity. Similarly, NK numbers per ml blood were reduced by laparotomy, but not by paw amputation [30]. Last, in patients subjected to rectosigmoid carcinoma excision, laparoscopy, compared to conventional excision, was shown to induce a significantly lower elevation in circulating IL-1β and IL-6 levels [130].

However, the clinical relevance of these results to cancer progression is unclear. When addressing disease free survival, several recent retrospective and prospective studies indicate that there are no significant differences between laparoscopy and open surgery [131,132,133,134]. When considering surgical options for breast cancer treatment, mastectomy correlated with improved overall survival when compared to lumpectomy, apparently because the entire malignant mass was removed [135]. Positive resection margins, which are evaluated during lumpectomy, are associated with an increased rate of local recurrence [136,137,138]. On the other hand, recovery from mastectomy is longer, and frequently involves morbidity of the arm including lymphedema [139] as well as wound infections [140]. Yet a few randomized trials reported no difference in the long-term outcome between mastectomy and breast conserving treatment after 18 years of follow-up [141,142]. We have studied this issue in two syngeneic tumor models of metastasis, and to our surprise no significant effects of adding laparotomy to apparently smaller surgical trauma were evident. Specifically, the addition of laparotomy did not increase recurrence-associated mortality rates following excision of B16 melanoma from mice hind paws [30]. Similarly, unpublished data from our laboratory indicated that the addition of laparotomy did not affect the number of experimental liver metastases in mice injected with the CT26 colon cancer cells through a smaller incision. It is our hypothesis that most surgical procedures needed for the removal of a primary tumor, including laparoscopy and lumpectomy, are sufficiently traumatic to induce neuroendocrine and immunological effects that profoundly impact resistance to tumor progression. The magnitudes of these effects *vis-à-vis* tumor progression reach ceiling levels, although more severe surgeries clearly cause more profound effects in other physiological indices. This hypothesis should be further studied.

## 6. Scheduling Surgery According to the Estrous Cycle

Some studies in breast cancer patients [143], but not other studies [144,145], reported an up to three-fold difference in long-term recurrence rates depending on the timing of the surgery within the menstrual cycle. These findings were independent of sex-hormones receptor status, and occurred mostly in lymph node positive patients [143]. However, the desirable menstrual phase or the size of the effects were inconsistent between studies [146]. Fluctuations of estradiol and progesterone levels along the estrous cycle were suggested to impact CMI competence and resistance to metastasis in humans, but only few studies systematically addressed potential mechanisms. To address this issue we studied NK activity and resistance to metastasis in rats using the MADB106 mammary adenocarcinoma line. To our surprise, we found that while the estrous cycle had minimal effects on both indices, it profoundly affected the NK-suppressive and metastasis-promoting effects of a β_2_-AR agonist and of surgery [147,148,149]. In addition we studied healthy women and found very similar effects: baseline levels of NK activity were not affected by the menstrual cycle, but NK cells were markedly more susceptible to suppression by a β_2_-AR agonist during the luteal phase compared to the follicular phase [148,150]. We ascribe the findings in both animals and healthy women to higher expression of β_2_-adrenoceptors on lymphocytes that was reported to occur during the luteal phase [151]. Because patients usually experience psychological and physiological stress, and thus elevated sympathetic activity, during the perioperative phase, these findings could underlie some of the reports in breast cancer patients.

In addition, human NK cells express intracellular estrogen receptors [152], and estrogen was shown to decrease NK cytotoxicity via its receptors [153]. *In vitro*, estradiol suppressed the cytotoxic activity of murine purified NK cells [154], and inhibited their IFNγ production [155]. Last, Wood *et al.* suggested that estrogen could promote the growth and metastatic spread of breast cancer cells through its ability to stimulate the production of VEGF and/or bFGF (basic fibroblast growth factor) by breast cancer cells [156].

Overall, although the clinical findings are inconsistent, neuroendocrine effects of catecholamines and sex hormones on tumor resistance should not be disregarded. If our findings have relevance to the clinical setting, then blocking sympathetic stress responses during the perioperative period may render the relations between timing of the surgery and long-term recurrence rate insignificant.

## 7. Using a Combination of Beta-Blockers and COX-2 Inhibitors in the Perioperative Context to Reduce the Risk of Metastatic Progression and Recurrence Rates

As elaborated above, prostaglandins and catecholamines are two key mediators of the deleterious effects of stress and surgery on the immune system and on tumor capacity to grow and metastasize. Therefore, preventing their synthesis or activity during the critical perioperative period would be beneficial, provided no clinically significant contraindications exist. Such a clinical approach seems feasible and easy to implement, as these drugs are already approved and used in the clinical context, including surgery to achieve other goals, and their side effects are few, if any. Specifically, beta-adrenergic blockers, such as propranolol, are used for several cardiovascular disorders, post-traumatic stress disorder, and some forms of anxiety, and their use during surgery is maintained [157]. COX-2 inhibitors, such as etodolac, are widely used to treat inflammation and pain, and are routinely employed during surgery in some hospitals [7,108]. Each of these families of drugs hold anti-cancer potential effects on both CMI and several types of cancer, including breast cancer, as described throughout this review.

Interestingly, we found that blocking only CA or only PG was insufficient, as the other un-inhibited hormone still suppressed immunity and promoted tumor development [23,30]. Specifically, our animal studies showed that the combined perioperative use of propranolol and etodolac attenuated metastatic progression, reduced cancer recurrence, and promoted disease-free survival rates [12,23,30,158], while each drug alone was significantly less effective or completely ineffective in these studies [12,23,30,158]. In mice and rats, this drug combination also prevented the suppression of NK cytotoxicity against syngeneic tumors, and attenuated other CMI perturbations [12,23]. The notion that the perioperative period is critical in controlling the metastatic process and determining survival rates is strengthened by the findings that even though these drugs were given during a short perioperative intervention period (up to 48 h), their effect was significant and long-lasting (studying long-term survival).

A complementary approach for overcoming postoperative immune suppression could be in the form of CMI enhancement before surgery, rather than or in addition to preventing surgery-induced immune suppression. Immunotherapeutic attempts unrelated to surgery have employed immunostimulatory agents as CpG oligodeoxynucleotides, poly I:C, and cytokines such as IL-2, or IL-12 and their combinations [159,160,161,162]. While animal studies showed encouraging results, many of the clinical treatments were associated with toxicity and side effects. Commonly, the administration of a single cytokine did not yield potent or durable effects. We were able to show in rats that continuous administration of a non-toxic doses of IL-12 or poly I:C (an IFNγ inducer) beginning five days before stress and surgery enhanced the number of circulating NK cells and/or their activity, and reduced mammary tumor colonization of the lungs [163]. Similarly, a single administration of the new immune stimulatory agent, CpG-C, the day before surgery, had similar beneficial impacts, without any side effects [164]. However, we also found that immunostimulation by IL-12 was rendered ineffective by the immunosuppressive effects of surgery [163]. Therefore, we recently combined the approaches of immune-enhancement with the use of beta blockers and COX-2 inhibitors, attempting to both strengthen CMI and prevent its suppression. Our recent results demonstrate that this integration of approaches has a synergistic effect in preventing the deleterious effects of surgery on immunity and tumor progression [158,164,165]. We further hypothesize that the use of β-blockers and COX-2 inhibitors simultaneously with immunostimulatory therapy would potentiate the efficacy of immunostimulatory agents by preventing some of their self-limiting effects [158,166].

Last, to address potential side-effects of propranolol and etodolac *vis-à-vis* tissue healing in the context of surgery, we recently tested in rats the impact of the perioperative use of these drugs on healing of the skin, muscle, and colonic anastomosis. We found no deleterious effects of these drugs in the doses we used to prevent immune suppression and limit the metastatic progression [167]. Other potential side effects of the proposed drugs could also be addressed in the context of surgery before conducting clinical trials.

## 8. Summary

Surgery is a necessary procedure for eradicating the primary tumor, and provides a window of opportunity for immunocytes to eliminate or gain control over minimal residual disease. However, surgery bears many risks leading to promotion of the metastatic process, through immune suppression, alteration of the tumor microenvironment, and direct impact on the malignant tissue. We believe that the key endocrine agents responsible for these deleterious effects of surgery are CA, PG, and potentially GC. Their levels increase as a result of perioperative psychological stress, as well as drug and anesthetic use and trauma-related stress responses. These agents promote tumor proliferation, invasion capacity, the release of pro-angiogenic factors, and reduce tumor anoikis. Moreover, these agents suppress many aspects of anti-tumor CMI through directly activating immunocyte receptors, by inducing the expression of inhibitory surface receptors on immunocytes, and by shifting the T_H_1/T_H_2 cytokine balance toward a T_H_2 dominance, all of which suppresses CMI.

Surgeons could benefit their patients by exploiting opportunity presented during the perioperative period to arrest the metastatic process and increase the chances of disease-free survival for their patients. As suggested by our animal studies, this could be achieved by introducing a short perioperative drug regimen that would eliminate the unnecessary excessive endocrine responses and prevent the above deleterious effects of surgery. We propose the perioperative administration of COX-2 inhibitors (such as etodolac) and β-AR blockers (such as propranolol), a combination that is relatively safe, easily applied, and can be used in most cancer patients. The drugs’ actions could reduce anxiety and stress, reduce morphine usage for pain alleviation, prevent CMI suppression by surgery and by PG-secreting tumors, and reduced the direct effects of CA and PG on the malignant tissue. The drug combination also has the potential to synergize with simultaneous use of immunotherapy. We believe that the integration of etodolac and propranolol into the preoperative setting of oncological surgery will improve postoperative well-being and decrease long-term recurrence rates in patients with breast, colon, and other types of cancer.
